# Polyamine Metabolism for Drug Intervention in Trypanosomatids

**DOI:** 10.3390/pathogens13010079

**Published:** 2024-01-16

**Authors:** Yolanda Pérez-Pertejo, Carlos García-Estrada, María Martínez-Valladares, Sankaranarayanan Murugesan, Rosa M. Reguera, Rafael Balaña-Fouce

**Affiliations:** 1 Departamento de Ciencias Biomédicas, Campus de Vegazana s/n, Universidad de León, 24071 León, Spain; myperp@unileon.es (Y.P.-P.); cgare@unileon.es (C.G.-E.); rmregt@unileon.es (R.M.R.); 2 Instituto de Biomedicina (IBIOMED), Campus de Vegazana s/n, Universidad de León, 24071 León, Spain; 3 Instituto de Ganadería de Montaña (IGM), CSIC, Universidad de León, 24346 Grulleros, Spain; mmarva@unileon.es; 4 Medicinal Chemistry Research Laboratory, Department of Pharmacy, Birla Institute of Technology and Science-Pilani, Pilani 333031, India; murugesan@pilani.bits-pilani.ac.in

**Keywords:** neglected tropical diseases, trypanosomatids, sleeping sickness, Chagas disease, leishmaniasis, polyamines, ornithine decarboxylase, S-adenosylmethionine decarboxylase, trypanothione, eflornithine (DFMO)

## Abstract

Neglected tropical diseases transmitted by trypanosomatids include three major human scourges that globally affect the world’s poorest people: African trypanosomiasis or sleeping sickness, American trypanosomiasis or Chagas disease and different types of leishmaniasis. Different metabolic pathways have been targeted to find antitrypanosomatid drugs, including polyamine metabolism. Since their discovery, the naturally occurring polyamines, putrescine, spermidine and spermine, have been considered important metabolites involved in cell growth. With a complex metabolism involving biosynthesis, catabolism and interconversion, the synthesis of putrescine and spermidine was targeted by thousands of compounds in an effort to produce cell growth blockade in tumor and infectious processes with limited success. However, the discovery of eflornithine (DFMO) as a curative drug against sleeping sickness encouraged researchers to develop new molecules against these diseases. Polyamine synthesis inhibitors have also provided insight into the peculiarities of this pathway between the host and the parasite, and also among different trypanosomatid species, thus allowing the search for new specific chemical entities aimed to treat these diseases and leading to the investigation of target-based scaffolds. The main molecular targets include the enzymes involved in polyamine biosynthesis (ornithine decarboxylase, S-adenosylmethionine decarboxylase and spermidine synthase), enzymes participating in their uptake from the environment, and the enzymes involved in the redox balance of the parasite. In this review, we summarize the research behind polyamine-based treatments, the current trends, and the main challenges in this field.

## 1. Introduction

Neglected tropical diseases (NTDs) are a group of parasitic diseases that affect more than a third of the world’s population but receive little or no attention from big pharmaceutical companies. Trypanosomatid-borne NTDs, sleeping sickness, Chagas disease and the various types of leishmaniasis are among the most neglected ones, thereby threatening the development of affected countries and compromising their development. These are zoonotic diseases transmitted by insect vectors that are becoming increasingly abundant in traditionally nonendemic areas due to climatic phenomena linked to global warming [1,2]. Despite decades of efforts, none of these diseases has an effective preventive vaccine, and treatment is based on health policy measures and pharmacological treatments. Unfortunately, most of the drugs used against these diseases are outdated, have numerous toxicity problems and undesirable side effects, require parenteral administration and some of them are even unstable and need a cold chain to be delivered at the point of care [3,4,5].

African trypanosomiasis or sleeping sickness is caused by *Trypanosoma brucei*—subspecies *gambiense* and *rhodesiense*—and affects populations in sub-Saharan Africa where the fly vector lives [6]. Thanks to WHO surveillance and control plans, more and more traditionally endemic African countries are declared free of sleeping sickness [7]. From an estimated 30,000 affected around the corner of the 21st century, the incidence of the disease has been reduced to <1 case per 10,000 population/year for five consecutive years in several regions of sub-Saharan Africa, being on the road to eradication [8].

American trypanosomiasis or Chagas disease is an endemic pathology caused by *Trypanosoma cruzi* in Latin American countries [9]. Poor hygienic conditions in the residences of people living in rural regions of endemic countries allow transmission of the parasite through the feces of hematophagous triatomine bugs [10]. However, as a consequence of increasing human migratory flows, there is an increasing number of documented cases in the southern United States, Canada, Europe, Japan and Australia [11]. According to WHO, an estimated 6–7 million people, mainly in 21 Latin American countries, are infected by the parasite, with an estimated incidence of 30,000 new cases per year and 12,000 deaths per year [7].

Finally, there are more than twenty species of the genus *Leishmania*, widespread globally with the exception of Oceania, which cause a group of diseases called leishmaniasis [3]. The three main forms of these diseases are cutaneous, mucocutaneous and visceral leishmaniasis. Cutaneous leishmaniasis (CL) is the least severe, rarely fatal, but stigmatizing form, as it produces disfiguring skin lesions that can be misidentified as leprosy. It is caused by several species of *Leishmania*—*L. major* and *L. tropica* in Old World countries and *L. mexicana*, *L. braziliensis*, etc., in many regions of Latin America [12]. Other species of the *L. braziliensis* complex, namely *L. panamensis* and *L. guyanensis*, are responsible for mucocutaneous leishmaniasis (MCL), a severe manifestation that causes deep mucosal lesions in the nose and mouth, thus resulting in disfigurement and stigmatization, and may even lead to metastasis [13]. Finally, the most severe and potentially fatal manifestation is visceral leishmaniasis (VL) caused by *L. infantum* and *L. donovani* (in this case, the transmission of the parasite is anthroponotic) [14]. VL is widespread in sub-Saharan East Africa (where most deaths are reported), Asia and the Indian subcontinent (where the highest cases of resistance to common drugs have been reported), Mediterranean Europe and South America (caused by *L. infantum chagasi*) [3,15]. According to the latest WHO report, during the period 2014–2022, the number of reported cases of VL has decreased by 59%, and the number of reported deaths is estimated at more than 4000, although this represents only a small percentage of the actual cases [7]. In addition, a high percentage of treated cases of VL gives rise to a relapsing form of the disease called post-kala-azar dermal leishmaniasis [16].

**Figure 1:**
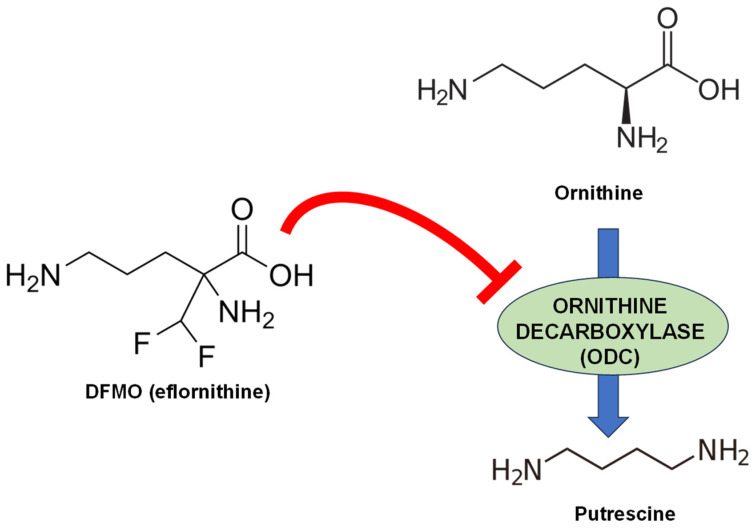
Chemical structure of DFMO (eflornithine) and mechanism of action as irreversible inhibitor of ornithine decarboxylase (ODC) in *T. brucei*.

The main concerns about treatments for trypanosomatid-transmitted NTDs are the limited number of drugs available, their safety, as well as the emergence of resistance problems due to overuse or misuse. Sleeping sickness is currently being treated with the combination therapy NECT, comprising nifurtimox and the ornithine analog α-difluoromethylornithine (DFMO; eflornithine) (Figure 1), and recently, fexinidazole has been successfully included [17]. For Chagas disease, the nitroheterocycles nifurtimox and benznidazole have been the two drugs of choice for several decades with good results [18].

The most difficult treatments are those intended to treat the different forms of leishmaniasis [19]. First-line drugs consist of pentavalent antimony derivatives, which have been used for almost one century. However, despite the hundreds of thousands of lives they have saved, they are obsolete medicines requiring parenteral administration, generally subcutaneous or intramuscular, daily dosing for several weeks and serious side effects that prevent their use in patients of certain ages [20]. Moreover, misuse has promoted the emergence of resistant strains in many regions [21]. Amphotericin B—under different presentations, including liposomal administration—is a very effective drug, but its poor oral bioavailability requires slow-infusion intravenous administration with hospital admission [22]. Miltefosine is the only oral drug with high efficacy against leishmaniasis. However, it can easily give rise to resistant strains and has embryotoxic effects [23]. As a control measure for VL in endemic African countries, several international agencies have suggested the combination of the above-mentioned drugs with the aminoglycoside antibiotic paromomycin [24].

## 2. Druggable Targets in Trypanosomatids

**Figure 2:**
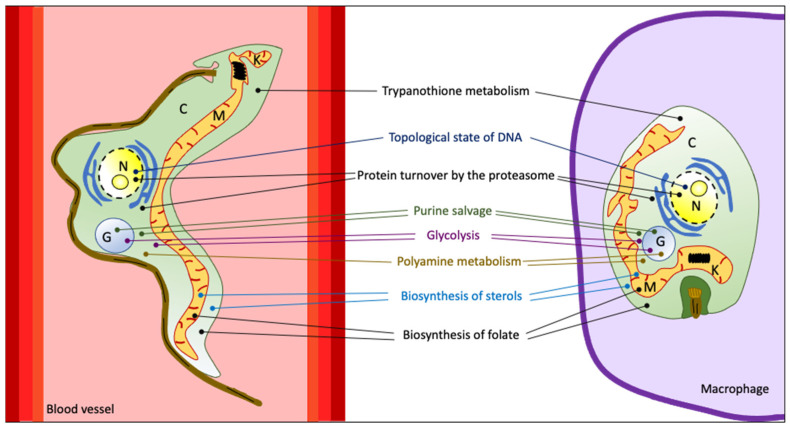
Schematic representation of a bloodstream *T. brucei* trypomastigote (**left picture**) and an intracellular *T. cruzi* and *Leishmania* amastigote (**right picture**). The major metabolic pathways that have been considered as potential drug targets are indicated. Some organelles are also represented: C: cytosol; G: glycosome; K: kinetoplast; M: mitochondrion; N: nucleus.

Finding and designing new promising antitrypanosomatid drugs is linked to the identification of specific proteins and enzymes in the parasite that can be targeted to control the growth and proliferation of these parasites. Therefore, biochemical characterization of different trypanosomatid-specific pathways that are essential for the viability of the parasite can shed light on the discovery of suitable drug targets. Several metabolic pathways in trypanosomatids (Figure 2) represent good candidates for this purpose, and this topic has been the subject of extensive recent revisions (for example [25,26,27]). Summarized information is indicated below.

### 2.1. Biosynthesis of Sterols

Sterols are terpene-derived molecules that are key structural components mainly found in the cell membrane, thus maintaining cellular structures and representing an important constituent for several cellular functions [28]. Unlike mammalian cells, where cholesterol is the main sterol, trypanosomatids contain ergosterol and 24-methyl sterol, which are major sterols essential for growth and viability. In fact, the primary site of action of the well-known antileishmanial drug amphotericin B is ergosterol, thereby disrupting the permeability barrier of the parasite membrane with subsequent ion leakage and cell death [29]. Therefore, the sterol biosynthetic pathway represents an attractive target for antitrypanosomatid drugs, and several enzymes of this pathway have been reported to be affected by different compounds [30]. For example, 3-hydroxy-3-methylglutaryl coenzyme-A reductase, catalyzing the conversion of 3-hydroxy-3-methylglutaryl coenzyme-A into mevalonate (the precursor molecule for the synthesis of ergosterol) is inhibited in *Trypanosoma* and *Leishmania* by statins [31,32,33], and sterol methyl transferase, the last enzyme in the ergosterol biosynthesis that is absent in the mammalian host, is inhibited by azasterols in *Leishmania* and *T. cruzi* [34] and by 26-fluorolanosterol in *T. brucei* [35].

### 2.2. Glycolysis

A distinctive feature of trypanosomatids is the fact that glycolysis is compartmentalized between the cytosol and the glycosome, a peroxisome-related organelle where the first seven enzymes converting glucose into 3-phosphoglycerate are located. Glycosomes are also involved in beta oxidation of fatty acids, purine salvage, pentose phosphate pathway, pyrimidine biosynthesis, ether lipid biosynthesis, squalene synthesis, and oxidative stress protection [36]. Interaction with glycolytic enzymes has been reported for some efficient antitrypanosomatids compounds, such as the polysulphonatednaphthylamine drug suramine (the current treatment of the hemolymphatic stage of African trypanosomiasis or sleeping sickness), which has been shown to bind and inhibit the *Leishmania* phosphoglycerate kinase [37], or the para-amidosulfonamide ML251, which behaves as a potent inhibitor of *T. brucei* and *T. cruzi* phosphofructokinase [38]. Differences in the structure of some *Leishmania* and *Trypanosoma* glycolytic enzymes with human counterparts make them important candidates for drug design [39,40,41]. For example, *Leishmania* enolase and aldolase (62% and 48% identity, respectively, with the human orthologs) are able to stimulate host Th1 proteins as a response to the *Leishmania* infection, thus making them interesting targets in the development of effective vaccines against visceral leishmaniasis [42,43].

### 2.3. Purine Salvage Pathway

Another peculiarity of trypanosomatids is the inability to synthesize the purine ring de novo, hence being absolutely dependent on exogenous purines obtained from hosts to meet purine demand. The purine salvage pathway takes place between cytosol and glycosomes and comprises several enzymes, such as three phosphoribosyltransferases, adenine phosphoribosyltransferase (APRT), adenosine kinase (AK), several purine interconversion enzymes, hypoxanthine-guanine phosphoribosyltransferase (HGPRT) and xanthine phosphoribosyltransferase (XPRT), although some differences have been reported between *T. brucei* and *Leishmania* in this pathway [44,45]. Therefore, the use of purine nucleoside analogs represents an effective approach to target the purine salvage pathway [46]. Allopurinol (a hypoxanthine analog) alone or in combination with other drugs showed efficacy against canine leishmaniosis [47], although its pharmacokinetic properties are a major limitation for the use of this compound in humans [48]. Inosine and adenosine analogs have also been used against *T. brucei* and *T. cruzi* [45].

### 2.4. Modification of the Topological State of DNA

During vital cell processes, such as DNA replication, transcription and repair, the compact DNA structure should be resolved to a more relaxed form, thus allowing different proteins to carry out their function on DNA. Control of the topology of DNA through the relaxation of supercoiled DNA is catalyzed by type I (ATP-independent enzymes that break one DNA strand) and type II (ATP-dependent enzymes that break both DNA strands) topoisomerases [49]. The relevance of these enzymes in cell viability, together with the important structural differences existing between parasite and host’s topoisomerases (mainly in the case of DNA topoisomerase I), make these enzymes potential targets for the discovery of molecules against trypanosomatids [50]. Topoisomerase inhibitors are classified into poisons (class I) and catalytic inhibitors (class II) [51,52], and several topoisomerase inhibitors have been assayed in trypanosomatids, including camptothecins, fluoroquinolones, berberin, acridines, etc. [25,53].

### 2.5. Biosynthesis of Folate

Due to the importance of folates (pteridine-derivative molecules) as methyl-group donors in crucial one-carbon pathways, including amino acid metabolism and nucleic acid synthesis [54], enzymes involved in the metabolism of folate represent potential targets for antileishmanial compounds. Since trypanosomatids are autotrophic for both folate and unconjugated pteridines, these molecules are obtained from hosts, and once inside the parasite, folates are reduced by a bifunctional dihydrofolate reductase thymidylate synthase (DHFR-TS) and by a pteridine reductase 1 (PTR1), the latter being able to reduce both folates and unconjugated pteridines [55]. Therefore, an approach to target folate production in trypanosomatids is the simultaneous inhibition of both enzymes [56,57,58].

### 2.6. Protein Turnover by the Proteasome

The turnover of damaged, abnormal and unwanted proteins is carried out by a multi-subunit enzyme complex comprising the proteasome. Kinetoplastid (including trypanosomatids) proteasomes are mostly similar to those of other eukaryotes, although distinguishing features have been reported [59]. The majority of proteins that are degraded by the proteasome are ubiquitinated, and both the proteasome and the ubiquitination machinery, which function together as well as separately, are essential for the survival of trypanosomatids. Therefore, they have become an attractive therapeutic target in diseases caused by trypanosomatids [60]. Selective inhibitors of kinetoplastid proteasome showing promising results both in vitro and in vivo against these parasites have been identified [61,62,63], thus suggesting that the proteasome might represent the Achilles’ heel of all trypanosomids [26].

### 2.7. Polyamine and Redox Metabolism

One of the main metabolites associated with cell viability and regulating growth and differentiation processes in trypanosomatids is polyamine. The more abundant polyamines in trypanosomatids are putrescine and spermidine (without back-conversion of spermidine to putrescine) since these parasites do not synthesize or utilize spermine (reviewed in [64]). In *Leishmania*, the biosynthesis of polyamines is compartmentalized between the glycosome and cytosol [65] and uses arginine as a precursor amino acid [66,67]. Growth and survival in trypanosomatids are critically dependent on spermidine, and this polyamine has been shown to play a critical role in cell proliferation in *L. donovani* [68]. 

Spermidine is also involved in the synthesis of hypusine in the translation initiation factor 5A (eIF-5A) [69], and trypanothione (N_1_, N_8_-bis(glutathionyl)spermidine) [70], the key player molecule involved in redox defense in trypanosomatids (*vide infra*). Therefore, polyamine and redox metabolism represent other important targets for antitrypanosomatid chemotherapy, and enzymes involved in these pathways are described below in detail, together with their role as chemotherapy targets for the treatment of trypanosomatid-borne diseases.

## 3. The Biosynthetic Core of Polyamines

**Figure 3:**
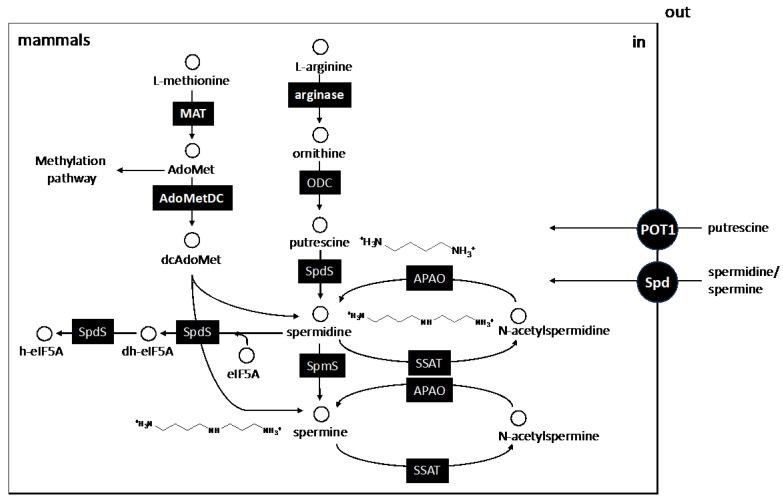
The canonic polyamine biosynthetic pathway of mammals (hosts of trypanosomatids). The enzymes involved in polyamine biosynthesis are shown in black boxes, and the metabolites are in empty circles. The full polyamine metabolic pathway includes biosynthesis and interconversion routes that starts from the essential amino acid L-methionine and from the semi-essential L-arginine. Putrescine, spermidine and spermine are synthesized from L-ornithine and AdoMetDc after decarboxylation, the activity of both enzymes being closely regulated by the cells. Both enzymes have been addressed as druggable targets for therapeutic intervention. Note that regardless of trypanosomatids, there is not any connection between polyamines and glutathione in mammals. Abbreviations of the enzymes and transporters and their corresponding EC numbers are given in alphabetic order: AdoMetDC, S-adenosylmethione decarboxylase (EC 4.1.1.50); APAO: N 1-acetylpolyamine oxidase (EC 1.5.3.13); Arginase (EC 3.5.3.1); DHS, deoxyhypusine synthase (EC: 2.5.1.46); DOHH deoxyhypusine hydroxylase (EC 1.14.99.29); MAT, methionine adenosyltransferase (EC 2.5.1.6); ODC, ornithine decarboxylase (EC 4.1.1.17); POT1, putrescine transport 1 (EC 7.6.2.16); SpdS, spermidine synthase (EC 2.5.1.16); SpmS, spermine synthase (EC 2.5.1.22). Abbreviations for the metabolites are also given in alphabetic order: AdoMet, S-adenosylmethionine; dcAdoMet, decarboxylated S-adenosylmethionine; eIF5A, eukaryotic translation initiation factor 5A; dh-eIF5A, deoxyhypusine-eIF5A; h-eIF5A, hypusine-eIF5A.

The natural polyamines putrescine, spermidine and spermine are small polycationic molecules synthesized from their precursor amino acids, L-arginine and L-methionine, and are involved in multiple metabolic processes, mostly related to cell proliferation. The metabolism of polyamines in trypanosomatids differs significantly from that of mammals (Figure 3) and presents a series of singularities among the three main trypanosomatid species. The most significant difference with respect to their hosts is the absence of spermine as the main polyamine and, consequently, of the gene encoding the enzyme responsible for its synthesis, spermine synthase. In addition, all enzymes involved in polyamine acetylation and oxidation (spermidine/spermine acetyl transferase and polyamine oxidase) that are present in the polyamine reconversion pathway in mammals are absent in these parasites (see review by [71]).

**Figure 4:**
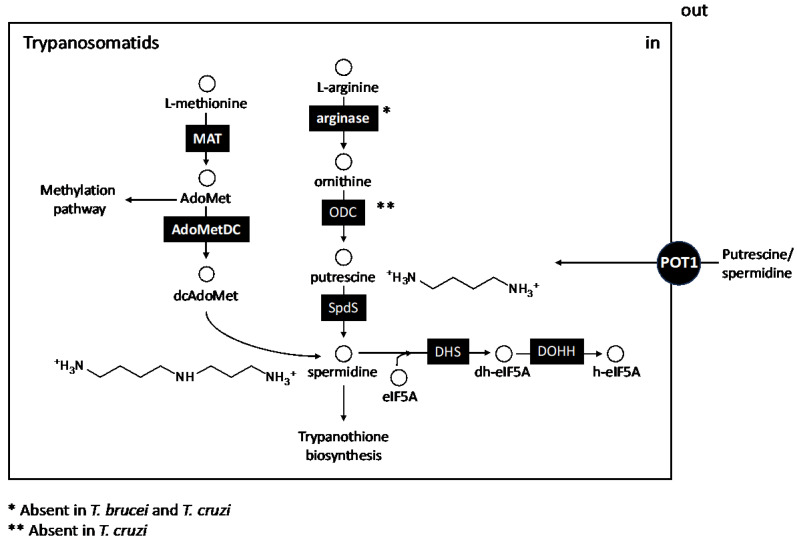
The polyamine biosynthetic pathway of trypanosomatid (African, American trypanosomes and *Leishmania*) is much simpler than the host pathway and lacks spermine and the whole interconversion pathway. However, it is in part devoted to ROS detoxification by means of the conjugation of spermidine to glutathione to form trypanothione (see Figure 3). Polyamine biosynthesis has some specific peculiarities involving: the lack of a true arginase in *T. brucei* and *T. cruzi* and the lack of the *odc* encoding gene in *T. cruzi* that make this parasite auxotrophic for putrescine and/or spermidine. Abbreviations of the enzymes and transporters and their corresponding EC numbers are given in alphabetic order: AdoMetDC, S-adenosylmethione decarboxylase (EC 4.1.1.50); Arginase (EC 3.5.3.1); DHS, deoxyhypusine synthase (EC: 2.5.1.46); DOHH deoxyhypusine hydroxylase (EC 1.14.99.29); MAT, methionine adenosyltransferase (EC 2.5.1.6); ODC, ornithine decarboxylase (EC 4.1.1.17); POT1, putrescine transport 1 (EC 7.6.2.16); SpdS, spermidine synthase (EC 2.5.1.16); Abbreviations for the metabolites are also given in alphabetic order: AdoMet, S-adenosylmethionine; dcAdoMet, decarboxylated S-adenosylmethionine; eIF5A, eukaryotic translation initiation factor 5A; dh-eIF5A, deoxyhypusine-eIF5A; h-eIF5A, hypusine-eIF5A.

Thus, the biosynthetic scheme of trypanosomatids is reduced to the synthesis of putrescine and spermidine from their precursor amino acids, including the specialized transporters in charge of importing these compounds. However, it is noteworthy that even this basic biosynthesis scheme is altered in *T. cruzi*, since this parasite lacks the gene encoding ornithine decarboxylase (ODC), and therefore, it is auxotrophic in putrescine (Figure 4). This fact justifies the inability of α-difluoromethylornithine (DFMO; eflornithine)—the irreversible inhibitor of ODC—to prevent the growth of this parasite in vitro and in vivo, unlike in *T. brucei* and in various *Leishmania* species [72]. These differences are even bigger with other parasites such as *Toxoplasma gondii*, which lacks the genes encoding putrescine and spermidine synthesis and the intracellular levels of these polyamines are solely dependent on transporters [73], or *Plasmodium* and other apicomplexans, which contain bifunctional enzymes capable of synthesizing simultaneously both polyamines [74].

In all species that retain the central core of polyamine synthesis, putrescine and spermidine are derived from two essential amino acids, L-arginine and L-methionine, respectively. Arginine undergoes enzymatic hydrolysis by arginase to yield ornithine, which is sequentially decarboxylated by ODC, a pyridoxal 5’phosphate (PLP)-dependent dimeric decarboxylase. ODC phylogenetically has a lysine residue at the interface of the cavity formed between the two monomers. Ornithine displaces lysine to form a Schiff base with PLP, thus decarboxylating the aldimine intermediate, which, upon reassembly, releases putrescine and returns the structure to its original conditions (PLP bound to the lysine residue) [75]. A unique feature of the mammalian ODC is its adaptability to the intracellular metabolic needs for polyamines, thus being induced and repressed very rapidly. This plasticity is conferred by a half-life of only 10–20 min, the shortest half-life of all known enzymes. This intricate regulatory mechanism is absent in trypanosomatids, whose turnover is much slower, thereby making ODC a differential pharmacological target compared to mammals. Associated with mammalian ODC, an anti-enzyme known as antizyme (AZ), which is responsible for mammalian ODC proteolytic degradation, has been described [76,77]. Furthermore, this enzyme contains a proline-glutamate-serine-threonine (PEST) motif at its C-terminal end that is responsible for the rapid proteasomal degradation of the ODC-AZ complex in a ubiquitin-independent system [78]. The ODC of trypanosomatids lacks the PEST region and no AZ-coding gene has been described so far.

ODC is a proven therapeutic target in *T. brucei*. Indeed, DFMO was approved by the FDA against the central nervous system phase of African sleeping sickness caused by *T. brucei gambiense* [79]. However, the clinical efficacy of this inhibitor is limited against *T. b. rhodesiense* [80], probably due to the more rapid turnover of its protein in this subspecies [81]. DFMO is also effective in killing *Leishmania* promastigotes [82]. Studies with Δ*odc* strains of *L. donovani* have shown that these parasites require ODC activity to maintain robust infection in mice [83].

The second conserved central enzyme of polyamine synthesis is S-adenosylmethionine decarboxylase (AdoMetDC), an enzyme that uses S-adenosylmethionine (AdoMet), a product of the enzymatic condensation of L-methionine with ATP by methionine adenosyl transferase (MAT), as a substrate. AdoMetDC is a pyruvoyl-dependent decarboxylase whose cofactor is derived from the autocatalytic cleavage of a proenzyme that generates the active enzyme and stabilizes the intermediate carbanion formed during the decarboxylation reaction. Human AdoMetDC is a homodimer, and both the cleavage reaction and the decarboxylation of AdoMet are stimulated by putrescine [84]. Like ODC, mammalian AdoMetDC has a short half-life (1–3 h) and is susceptible to proteasomal degradation after ubiquitination.

A singularity of trypanosomatid AdoMetDC is its heterodimer structure with a catalytically inactive regulatory subunit known as prozyme. Genomic analysis of the two types of AdoMetDC genes present in trypanosomatids (orthologs to functional enzyme and prozyme, respectively) indicates that the gene encoding the prozyme is the result of genetic duplication of an ancestral gene encoding AdoMetDC, although the products of both genes share only 30% of the amino acid sequence [85]. Regulation of prozyme expression occurs at the translational level and levels of spermidine or putrescine do not correlate with changes in prozyme production. However, prozyme levels increase in response to loss of AdoMetDC activity in *T. brucei* [86,87,88]. A correlation has been found between dcAdoMet levels and prozyme expression in *T. brucei*, suggesting that this metabolite may regulate its translation into an active protein [88].

The N-terminal domain of trypanosomatid AdoMetDCs contains a 16–20 amino acid region that does not exist in other eukaryotic homologs. This motif is involved in activation by prozyme [89] through an allosteric mechanism involving conformational changes at the N-terminal end. Thus, X-ray structures of monomeric AdoMetDC from *T. brucei* and functional AdoMet/prozyme heterodimer indicate that the monomer is inactive due to autoinhibition by its N-terminal sequence. Heterodimerization with prozyme displaces this sequence from the active site by a complex mechanism [90]. Other studies in *T. brucei* have found evidence for a feedback mechanism in which AdoMetDC and dcAdoMet act as suppressors of prozyme translation. Upregulation of the prozyme is observed after depletion of dcAdoMet, the latter being caused by inhibition of AdoMetDC activity, inhibition of AdoMet synthase activity, or L-methionine depletion [91].

In the final step of polyamine biosynthesis, spermidine synthase (SpdS) catalyzes the enzymatic conjugation of the aminopropyl group of dcAdoMet with putrescine to give rise to spermidine. The crystal structure of the human SpdS dimer has been studied with a multisubstrate inhibitor S-adenosyl-1,8-diamino-3-thioctane (AdoDATO), and the products have shown that the active site, formed between the N-terminal domain and the central core, contains multiple residues that are highly conserved in other known aminopropyltransferases [92].

The regulation of SpdS in *T. brucei* is influenced by AdoMetDC activity, the latter enzyme being the key point in polyamine synthesis [86]. Attempting to validate SpdS as a target to treat African trypanosomiasis, RNAi experiments on the blood-borne form of *T. brucei* showed that *spdsyn* gene silencing correlates well with a decrease in intracellular spermidine levels and cessation of growth. This indicates that *T. brucei* is unable to obtain sufficient spermidine from its environment to achieve growth requirements [93]. The Δ*spdsyn* mutant strain of *L. donovani* requires spermidine to grow as promastigotes; and mouse infections performed with this strain demonstrated that SpdS activity is essential to maintain robust infection [94]. The relationship between AdoMetDC and SpdS is metabolically so close that it has been suggested that the two enzymes could form a heteromer in *L. donovani* [95].

## 4. Transport of Polyamines

Polyamine transport involves unique uptake and export mechanisms. The polycationic nature of polyamines at physiological pH, entails the need for transporters for their influx. Polyamine uptake is a net energy-consuming and saturable process that transports polyamines against concentration gradients. Separate transporters for putrescine, spermidine and spermine have been identified in mammalian cells, and uptake and excretion are mediated for each polyamine by the same transporter. Polyamine transport is fully integrated into the regulatory system controlling polyamines in mammalian cells [96]. In general, factors that increase the formation of polyamines enhance their uptake from the extracellular environment, and, conversely, situations in which there is an excessive accumulation of intracellular polyamines favor their elimination. Several proteins involved in the regulation of polyamine transport adjust the levels of polyamines in the cell. Thus, AZ has been found to negatively regulate polyamine transport [97].

The viability of trypanosomatids with auxotrophy in polyamine synthesis and the lack of efficacy of certain treatments based on polyamine synthesis have been attributed to polyamine transport mechanisms [98]. Given the inability of *T. cruzi* to synthesize putrescine due to the lack of the gene encoding ornithine decarboxylase, the intracellular availability of polyamines in this parasite depends entirely on transport processes. TcPOT1.2 is a high-affinity putrescine spermidine permease belonging to the amino acid-polyamine-organocation (APC, TC 2.A.3) family of transporters, with no mammalian homologs, according to the Transport Classification Database (TCDB, http://www.tcdb.org/) [99,100]. Overexpression of TcPOT1.2 in *T. cruzi* epimastigotes has shown greatly enhanced resistance to hydrogen peroxide and the trypanocidal drugs nifurtimox and benznidazole [101]. The differences between TcPOT1.2 and its mammalian counterparts make it an interesting therapeutic target for the search for inhibitors of this permease with trypanocidal activity [102]. L-ornithine uptake in this parasite is mediated by the TcCAT1.1 transporter, which binds with high affinity to L-arginine and low affinity to L-ornithine [103].

Two amino acid transporters, TbAAT10-1 (selective for ornithine), and TbAAT2-4, which transports ornithine and histidine, have been described in *T. brucei*. The uptake of ornithine by TbAAT2-4 is dependent on the histidine concentration in the extracellular medium, and the ornithine uptake performed by both transporters is responsible for the decrease in the potency of eflornithine [104]. As already mentioned, *T. brucei* cannot scavenge sufficient spermidine from their environment, which indicates the absence of an effective transporter for spermidine in this parasite [93]. A high-affinity transporter for putrescine has not been identified either [72].

The polyamine transporter POT1 from *L. major* was cloned and expressed in *Xenopus laevis* oocytes and showed a high affinity for both putrescine and spermidine. This member of the APC family is inhibited by several antileishmanial drugs with diamidine structure [105,106].

## 5. Other Polyamine-Linked Pathways

In addition to putrescine and spermidine synthesis, polyamines serve as a link to other important metabolic pathways in trypanosomatids that may be the subject of drug intervention. A brief summary of some of them and their peculiarities, if any, among trypanosomatids is discussed below.

### 5.1. S-Adenosilmethionine Synthesis: Transulfuration and Transmethylation Pathways

L-methionine is an essential proteinogenic amino acid and one of the precursors of polyamine synthesis in both trypanosomatids and their hosts. As mentioned above, after condensation with ATP to synthesize S-adenosylmethionine (AdoMet) and subsequent decarboxylation, it serves as a donor of aminopropyl groups for spermidine synthesis. AdoMet is a metabolite that represents an important metabolic crossroad because it also serves as a donor molecule for methyl groups in transmethylation reactions and for sulfo groups in transulfuration pathways. The transformation of L-methionine to AdoMet is carried out by the enzyme methionine adenosyltransferase (MAT). MAT is a dimeric enzyme that catalyzes the synthesis of AdoMet in two sequential steps; AdoMet formation and subsequent tripolyphosphate (PPPi) cleavage, a reaction induced by AdoMet [107].

*L. infantum* and *L. donovani* MAT-encoding genes have been cloned, and the enzymes characterized, including the identification of the aminoacids related to the active site [108,109,110]. Unlike the mammalian enzyme, *Leishmania* MAT activity is weakly regulated by AdoMet [108]. MAT-overproducing *L. donovani* promastigotes control AdoMet production, keep intracellular AdoMet concentration at levels that are compatible with cell survival, and do not show deleterious hypermethylation levels [111]. MAT activity and abundance vary throughout the culture time, thereby suggesting the presence of post-transcriptional regulatory mechanisms. Thus, it has been shown that this enzyme is proteolytically degraded by the *Leishmanial* proteasome [112].

### 5.2. Synthesis of Hypusine

The eukaryotic translation initiation factor 5A (eIF5A) is the only protein post-translationally modified with hypusine, an amino acid derived from the fusion of 2-hydroxyspermidine with the omega amino group of Lys. Modification of this factor is carried out by the sequential action of deoxyhypusine synthase (DHS), which, in a first extent, establishes a covalent intermediate between spermidine and a Lys residue at the DHS catalytic site. This intermediate is transferred to an eIF5A-specific Lys residue, which is sequentially oxidized by deoxyhypusine hydroxylase (DOHH) [113] (Figure 4). 

The human DHS is a homotetramer with four active sites located within deep tunnels and four NAD+ binding sites per tetramer [114]. In contrast, the genomes of Trypanosoma, *Leishmania* and *Entamoeba* encode two DHS paralogs [115,116,117]. The active form of *T. brucei* is a heterotetramer formed between a catalytically defective protein containing the catalytic residue Lys, and a DHS paralog where the Lys of the active center is replaced with Leu. Oligomerization of the two paralogs increases the activity of the first subunit alone, with each heterodimer interface containing a catalytic site and an inactive site [117]. Thus, to facilitate the identification and development of new DHS inhibitors, the two paralogs of *L. major* have been expressed in Saccharomyces cerevisiae mutant cells lacking the endogenous DHS. Ectopic expression of both LmDHS paralogs can rescue yeast mutant cells, whereas independent expression of both paralogs did not produce viable cells [118]. These structural differences could be exploited to design new inhibitors in the development of target-based therapies. The *L. donovani* DOHH encoding gene contains 981 base pairs and includes eight HEAT tandem repeats and four His-Glu sequences at the iron metal coordination sites [119]. A functional recombinant DOHH protein from *L. donovani* has been successfully targeted with metal chelators such as olamine cyclopirox and mimosine, which were more effective against the parasite enzyme than against the human enzyme [69], thus pointing to DOHH as a promising drug target against *Leishmania* [120].

### 5.3. Parasite Arginase/Host NO Synthesis Interplay

Trypanosomatids, like their hosts, are auxotrophic for L-arginine, which must be obtained from the diet. The main amino acid transporter of these parasites belongs to the AAAP (Amino Acid/Auxin Permease) family. AAAP family members have been identified and characterized from *L. donovani* and *T. cruzi*. These high-affinity, L-arginine-specific transporters are regulated by the metabolic availability of the amino acid [121,122,123,124]. Intracellular L-arginine has different functionalities, including polyamine biosynthesis. Arginase catalyzes the hydrolysis of L-arginine to produce urea and ornithine in the host, but this activity is not present in all trypanosomatids. Thus, arginase activity has been detected in *Leishmania* but not in *T. cruzi* or *T. brucei*, although the latter encodes an inactive arginase-like protein that lacks key catalytic residues [66,72,125]. For this reason, it is believed that the primary source of L-ornithine in *T. brucei* is synthesized by the host [126].

**Figure 5:**
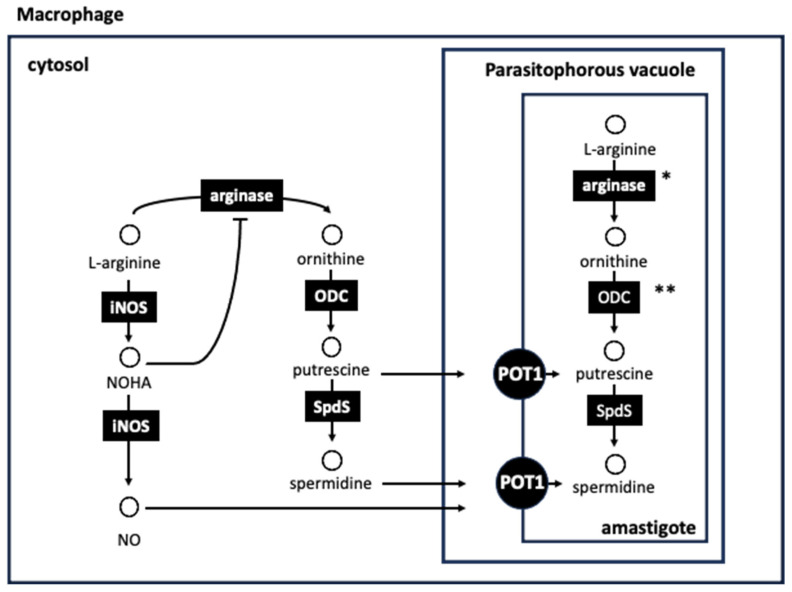
Host–parasite interplay in *Leishmania* infections. L-arginine plays a key role in macrophage activation and parasite survival during infection. Within the host macrophage, L-arginine is substrate of inducible nitric oxide synthase (iNOS) and arginase. iNOS/arginase balance is transcriptionally controlled by interleukins and is modulated at a biochemical level too. On the one hand, the classic M1 proinflammatory activation of macrophages responds to Th1 cytokines such as TNFa and IFNg and IL1, IL2 and IL10 interleukins. Alternative M2 anti-inflammatory activation of macrophages responds to Th2 cytokines such as TGFb, IL4, IL10 and IL13. M1 activation induces iNOS in macrophages, responsible for L-arginine cleavage to NO and citrulline byproduct. NO will promote the cascade production of nitrogen reactive species (RNOS) such as peroxynitrite radical (ONOO-). On the other hand, L-arginine cleavage by iNOS is a two-step enzymatic process that produces an important intermediate *N*-hydroxy-L-arginine (NOHA), which, before its complete hydrolysis to NO, can interfere arginase activity, preventing L-ornithine and polyamine production. Th2 response implies an increase in arginase activity, resulting in the formation of L-ornithine and polyamines, which can be used by the parasite. Inside the parasitophorous vacuole, *Leishmania* amastigotes can obtain L-arginine and polyamine from the host using the corresponding active transporters. *Leishmania* can synthesize putrescine and spermidine from L-arginine and L-methionine, which are essentials for the parasite, but *T. cruzi* is auxothroph for putrescine since it lacks genes encoding for both, a true arginase and ODC. * Absent in *T. brucei* and *T. cruzi*; ** Absent in *T. cruzi*.

In addition to its role as a component of proteins and as a precursor for polyamine biosynthesis, L-arginine has different functions in the host and the parasite. In the mammalian host, L-arginine is involved in ammonia detoxification via the urea cycle and in the production of nitric oxide (NO) mediated by inducible nitric oxide synthase (iNOS). NO is an important metabolite related with microbicidal mechanisms. Trypanosomatids directly remove ammonia, thus making most of the enzymes of the urea cycle unnecessary. Thus, L-arginine in trypanosomatids is mainly required to provide polyamines during parasite proliferation [66] (Figure 5).

*Leishmania* lives inside mammalian host macrophages, thus escaping from mechanisms of the immunological system, such as NO production by iNOS after macrophage Th1 activation that results in parasite killing [66]. In macrophages, L-arginine is the substrate for arginase and iNOS, and therefore, the amount of this amino acid available for both pathways is critical for parasite replication. Characterization of arginase null mutants in *L. mexicana* showed that the enzyme is essential for promastigote viability. Unlike mammalian cells, *Leishmania* produces only one protein with arginase activity. This protein is located inside glycosomes and provides polyamine precursors for the parasite [65]. Arginase null mutants of this parasite were able to establish infections in mice, albeit with lower levels of infectivity compared to wild-type parasites [127,128,129,130]. These findings show that amastigotes are able to uptake ornithine and polyamines from the phagolysosome, although full parasite arginase activity is required for maximal infection [130].

Studying the transcriptomic profiling of *L. amazonesis* wild-type and *L. amazonesis* arginase KO mutants revealed that the expression of a gene encoding an oxidoreductase-like protein was upregulated in the null mutant promastigotes compared to wild-type promastigotes, and in wild-type axenic amastigotes compared to wild-type promastigotes. The conserved domain composition observed in this oxidoreductase would indicate that it could act as a NO synthase-like (NOS-like), catalyzing the conversion of L-arginine and molecular oxygen into L-citrulline and NO. This work suggests that the NOS-like expression in *Leishmania*, could be related to metacyclogenesis and amastigotes growth and would be being regulated by the internal pool of L-arginine and arginase activity [131].

### 5.4. Synthesis and Redox Metabolism of Trypanothione

The most remarkable discovery about polyamine metabolism in trypanosomatids was its relationship to the ROS scavenging system. In mammals, the glutathione sulfur redox cycle is maintained by two enzymes; glutathione peroxidase (Gpx) which breaks down ROS by oxidizing reduced glutathione (GSH) to the oxidized form (GSSG), and glutathione reductase (Grd), which, in turn, is reduce by GSSG to maintain the GSH/GSSG ratio. However, this system is replaced in trypanosomatids by trypanothione T(SH)2, a dithiol comprising composed of two glutathione molecules linked by a spermidine bridge [132]. T(SH)2 replaces GSH in all trypanosomatids, and a redox cycle is established between T(SH)2 and the oxidized form TSST [133].

**Figure 6:**
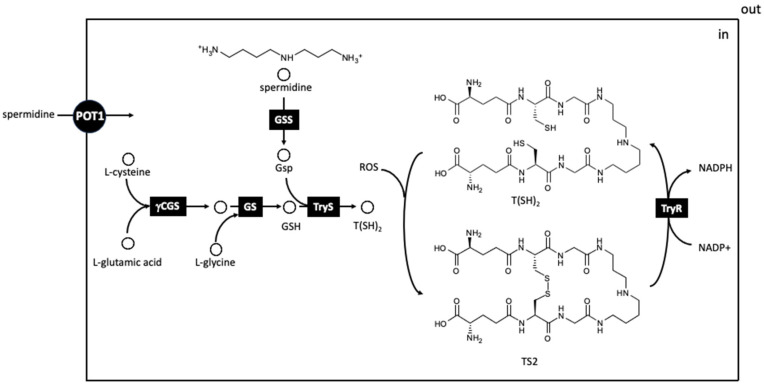
Trypanothione biosynthetic pathway and redox balance in trypanosomatids. Reduced trypanothione is a complex formed by two molecules of reduced glutathione bridged by their glycine residues with spermidine. Trypanothione can be oxidized by ROS to the oxidized form, establishing a sulfur redox balance enzymatically controlled by trypanothione reductase. The singularity of this ROS scavenger in trypanosomatids is an interesting druggable target for drug intervention in trypanosomatids. Abbreviations of the enzymes and transporters and their corresponding EC numbers are given in alphabetic order: *γ*GCS, *γ*-glutamylcysteine synthetase (EC 6.3.2.2); GS, glutathione synthase (EC 6.3.2.3); GSS, glutathionyl spermidine synthetase (EC 6.3.1.8); TryR, trypanothione reductase (EC 1.8.1.12); TryS, trypanothione synthase (EC 6.3.1.9). Abbreviations for the metabolites are also given in alphabetic order: *γ*GC, *γ*-glutamylcysteine; Gsp, glutathionylspermidine; GSH, reduced glutathione; ROS, reactive oxygen species; T(SH)2, reduced trypanothione; TS2, oxidized trypanothione.

The synthesis of T(SH)_2_ in *T. brucei*, *T. cruzi* and *Leishmania* spp. involves a two-step enzymatic condensation between GSH, which, in turn, binds to a spermidine molecule and then to a second GSH unit, which is carried out by a single enzyme trypanothione synthetase (TryS) [134]. Since T(SH)2 is required to detoxify ROS produced by host cells to destroy the intracellular parasite during infection, trypanosomatids encode an NADPH-dependent trypanothione reductase (TryR) in charge of reducing TSST and have been thoroughly studied as a potential target for drug intervention [135]. TryR is a homodimer that uses NADPH as an electron donor to the substrate binding domain or interface, which contains a cysteine disulfide in the active site and a FAD prosthetic group for electron transport [136]. TryR is a valuable drug target against Trypanosomatids, as it is absent in mammals, and the equivalent enzyme in the host, Grd, is different enough to design selective drugs [137]. In fact, disturbing TryR gives rise to toxic intermediate accumulation and oxidative stress, eventually causing parasite death [138] (Figure 6).

Due to the specificity of this pathway, it has been the target for many compounds, and although it is not within the scope of this review, some of them are briefly mentioned. Nitrofurans, which have been reported to act as reversible inhibitors of TryR [139] and nifuratel, a nitrofuran derivative, showed a time-dependent inhibitory effect on *L. donovani* TryR, likely acting as a “subversive” substrate, with good in vitro and in vivo antileishmanial effects in combination with miltefosine [140]. Different inhibitors of TryR have been tested against *Leishmania* [141,142] and *Trypanosoma* [143,144]. TryS has also been targeted by several inhibitors with antiparasitic activity, such as N5-substituted paullones and other small molecules identified by high throughput screening [145,146,147].

Since trypanosomatids lack the genes encoding both catalase and Gpx, an alternative antioxidant system must be in place to eliminate ROS produced by host macrophages.

Tryparedoxin (TXN) is a trypanosomatid-specific thioredoxin-like protein containing two cysteines at the amino-terminal end that can be either in reduced (TXN (red.) or oxidised (TXN (ox.) form. TXN peroxidase (TXPx) reduces various types of peroxides using electrons donated either directly from trypanothione, or via the redox intermediate TXN [148]. In a first step, TXPx reduces ROS substrates using electrons donated by TXN (red.), whose cysteines establish a disulfide bridge. To restore TXN (red.), an electron is donated by T(SH)_2_, which is in turn oxidized to TS. All of these enzymes have been proposed as attractive targets for drug discovery because of their unique presence in trypanosomatids and their absence in the mammalian host [149]. All enzymes involved in the antioxidant redox system are considered good drug targets in trypanosomatids, as deletion of the two alleles of their coding genes results in nonviable phenotypes unless the parasite is episomally transfected with the corresponding cloned genes [136].

## 6. Inhibitors of Polyamine Synthesis in Trypanosomatids

**Figure 7:**
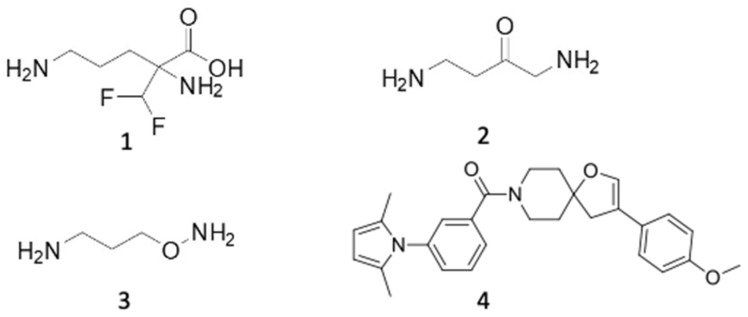
ODC inhibitors. DFMO (**1**) and other different chemical scaffolds inhibiting *Trypanosoma* ODC.

Since the late 1970s, polyamine metabolism has been identified as an important target for intervention in proliferative pathological processes, and numerous compounds have been synthesized and tested against different types of cancer [150]. Two anticancer compounds, an AsoMetDC inhibitor, methylglyoxal bisguanylhydrazone (MGBG) [151] and difluoromethylornithine (DFMO), an ODC inhibitor synthesized later on [152], were successfully tested against experimental trypanosome infections. The introduction of polyamine biosynthesis as a putative pathway for chemotherapy was due to the seminal work of Bacchi et al. in 1980 [153], who demonstrated the trypanocidal effect of DFMO in murine *T. brucei* infections. This work represents the flagship of all subsequent research in this field. In this chapter, given the immense number of compounds tested against the enzymes involved in the biosynthetic pathway and transport, we point out some of the most promising compounds or scaffolds against each of the targets mentioned in the previous sections (Figure 5, Figure 6 and Figure 7).

### 6.1. ODC Inhibitors 

DFMO (also known as eflornithine) (Figure 7) (**1**) is the best-known irreversible inhibitor of ODC and was synthesized to act as a suicide substrate in the active site of the enzyme by establishing a stable covalent addition to the protein, which leads to its irreversible inactivation. Many types of tumors, bladder, brain, esophagus, gastrointestinal tract, lung, oral cavity, mammary glands, stomach, skin and trachea (reviewed in [154]) have been effectively blocked with DFMO in animal models, but unfortunately, neither DFMO nor its analogs have gained approval for clinical application by FDA as single drug—based on its low toxicity—although it conserves considerable potential in cancer chemoprevention [155,156]. Recently, promising phase-II trials have been carried out in neuroblastoma with DFMO that may restart a new era for this compound in the cancer field [157]. However, since 1980, when Bacchi et al. found a curative effect of DFMO in experimental *T. brucei* infections in mice, expectations for this antiparasitic compound have been raised [153]. 

Fairlamb and coworkers showed that DFMO produced a marked increase in intracellular levels of polyamine precursor amino acids, such as ornithine, and a concomitant decrease in putrescine levels to negligible levels, and a reduction in spermidine amounts by 76% in *T. brucei*. In addition, depletion of polyamines led to a reduction in trypanothione synthesis, and ultimately, it resulted in a selective cytotoxic effect on the parasite [158]. DFMO is the only polyamine synthesis inhibitor approved by FDA that has a curative effect against *T. brucei gambiense* (but not *rodhesiense*) within the so-called NECT in the neurological stages of sleeping sickness in Africa in combination with the nitrofuran derivative nifurtimox [159,160]. The persistent inhibition of DFMO on the ODC of the parasite, but not in the host due to differences in intracellular turnover rates, leads to a blockade of spermidine and trypanothione synthesis, which renders the parasite vulnerable to detrimental free radical scavenging [161].

DFMO is a fairly safe drug with almost no toxic effects, but it has serious pharmacokinetic problems, such as the need for high doses and its low oral bioavailability, which requires intravenous administration of large infusion volumes [79]. Furthermore, it should be noted that ODC is not a target in *T. cruzi*, due to the absence of the gene encoding the enzyme in this parasite. In *Leishmania*, although ODC has been shown to be a pharmacotherapeutic target by genetic studies [162], the efficacy of DFMO and analogs is just observable in free-living forms, promastigotes [82], but this compound failed in intramacrophagic amastigotes and in vivo infections [163]. Other drug scaffolds to target ODC have been developed, some displaying ornithine (**2**) or putrescine (**3**) analogies [164,165] or owing to the structural dissimilarity between the ODC of humans and *L. donovani* (**4**), with different results [166].

### 6.2. AdoMetDC Inhibitors

**Figure 8:**
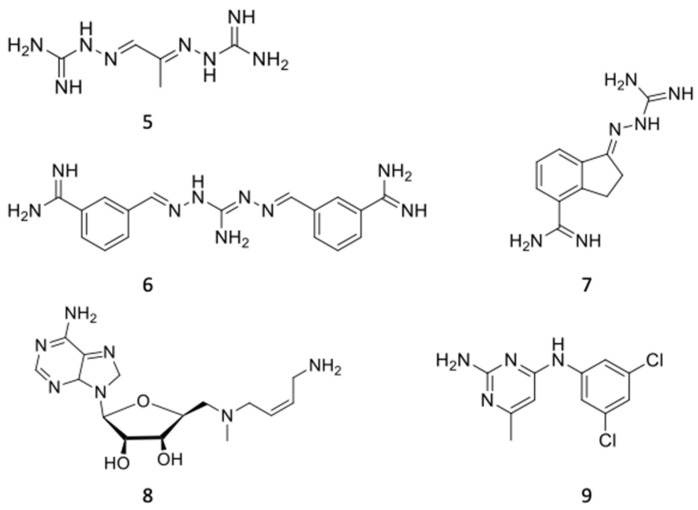
Polyamine uptake inhibitors. MGBG (**5**), the diamidines CGP40215A (**6**) and CGP48664A (**7**), MDL 73811 (**8**), UTSam568 (**9**).

AdoMetDC was also initially considered a potential anticancer drug target [151]. However, very few compounds reached clinical phase trials, and none of them are currently in clinical use [167]. As for ODC, the essentiality of AdoMetDC was genetically demonstrated in several trypanosomatids, and different small molecules were developed for pharmacological intervention in the three main trypanosomatids, as the AdoMetDC gene is present in all three species [168]. A first generation of AdoMetDC inhibitors was developed, including derivatives carrying guanidino groups, such as methylglyoxal bis(guanylhydrazone) (MGBG). MGBG (Figure 8) (**5**) showed antitrypanosomal activity against procyclic forms of *T. brucei* in vitro but failed in mouse models [169]. Similarly, despite its strong antileishmanial activity on promastigotes, it also failed against intramacrophagic amastigotes of *L. donovani* [170].

Second-generation AdoMetDC inhibitors, with a structure that resembles MGBG, include aromatic diamidines with known antiparasitic efficacy, such as berenil and pentamidine [171]. These diamidines proved to be effective reversible inhibitors of AdoMetDC and, in addition, inhibitors of polyamine uptake, thus showing an interesting inhibition profile of two different processes [172]. The diamidines CGP40215A (**6**) and CGP48664A (**7**) (Figure 8) were effective in curing laboratory infections by *T. brucei brucei*, *T. brucei rhodesiense*, *T. brucei gambiense* and the veterinarian strain *T. congolense* when used alone or in combination with DFMO [173]. This compound was a potent inhibitor of the growth of extracellular forms of *L. donovani* and *T. cruzi* in vitro, but failed against intramacrophage infections [170,174]. More recently, other novel diamidines have been assayed against the agents of animal trypanosomiasis *T. congolense* and *T. vivax*, showing curative outputs in murine models [175].

Third-generation AdoMetDC inhibitors are adenosine-based drugs similar to dcAdoMet. MDL 73811 (**8**) (Figure 8) is a potent irreversible inhibitor of *T. brucei* AdoMetDC and parasite growth, leading to the cure of the hemolymphatic phase of trypanosomiasis caused by *T. b. brucei* [176,177]. However, MDL 73811 and its methyl derivative Genz-644131 [178] were not effective in a murine model of the late phase with central nervous system involvement due to their poor permeation through the blood–brain barrier [179]. Other adenosine analogs similar to the polyamine byproduct methylthioadenosine were synthesized and showed significant activity both in vitro and in vivo against *T. brucei rhodesiense* species [180]. 

Recently, several HTS approaches, including repurposing of old drugs [181], have been developed to find new AdoMetDC inhibitors. Volkov et al. focused on the development of new therapeutics containing pyrimidinamine as a pharmacophore targeting the *T. brucei* AdoMetDC [182]. Using a high-throughput target-based screening, compound **9** (UTSam568) (Figure 8) was identified for its selectivity for *T. brucei* AdoMetDC, its inhibition of parasite growth and its predicted good penetration of the blood–brain barrier [183].

### 6.3. Inhibition of Spermidine Synthase

Unlike ODC and AdoMetDC, spermidine synthase has attracted little interest among researchers, although this enzyme has been genetically demonstrated to be a suitable target [94]. Only cyclohexylamine and S-adenosyl-1,8-diamino-3-thioooctane (AdoDato) have been shown to effectively inhibit the enzyme in *T. brucei* and cause its proliferative arrest in *T. brucei* [184]. In *Leishmania*, n-butylamine is able to inhibit this enzyme, and it may also act on ODC [185]. It has been speculated that the relatively large size of the active site of SpdS could represent a problem for inhibitor design [186].

### 6.4. Inhibitors of Polyamine Uptake 

**Figure 9:**
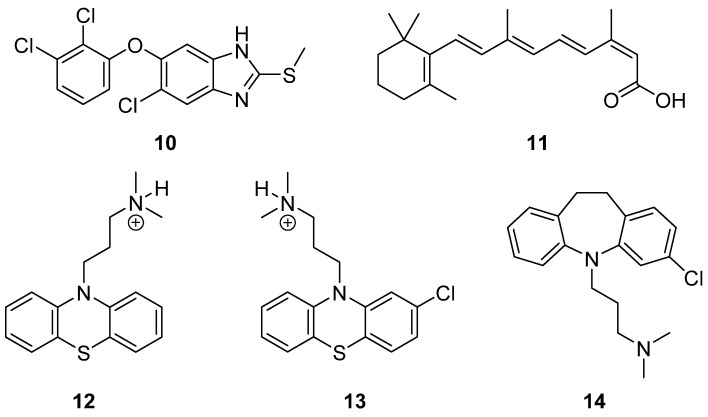
AdoMetDC inhibitors. Triclabendazole (**10**), isotretinoin (**11**)**,** promazine (**12**), chlorpromazine (**13**) and chlomipramine (**14**).

The polyamine auxotrophy of *T. cruzi* and the intramacrophagic location of *Leishmania* parasites, where DFMO inhibition can be circumvented by a high-affinity and inducible polyamine transporter, make polyamine transport an attractive drug target, in contrast to *T. brucei*, where ODC inhibition causes parasite death and may explain the curative effect of late-stage sleeping sickness in combination with nifurtimox [98]. As stated previously, aromatic diamidines, namely pentamidine or berenil, inhibited putrescine uptake—in addition to inhibiting AdoMetDC, thus causing polyamine depletion and cell death in *Leishmania* promastigotes [172]. Since then, polyamine analogs have been designed to inhibit polyamine uptake in combination with inhibitors of polyamine synthesis in order to deprive parasites of external supply. The first generation of these compounds consisted of symmetrically or nonterminally alkylated polyamine analogs [187]. Substituted polyamine-based derivatives have also been identified, including diamines and imidazole-containing drugs like triclabendazole (comp 10), which showed a multisite mode of action in the parasite [188,189,190] (Figure 9).

Several drug screenings have been carried out to identify novel polyamine uptake inhibitors against *T. cruzi* [190]. A virtual screening of several FDA-approved drugs [191] identified isotretinoin (compound **11**) (Figure 9) as the most potent compound with IC_50_ in the low micromolar range for the inhibition of putrescine uptake in T. cruzi. Further investigation was carried out to validate the trypanocidal activity of this compound by testing its efficacy in inhibiting trypomastigote and epimastigote stages of T. cruzi. The same group in 2019 made use of a drug repurposing strategy to identify molecules with similar phenothiazines and dibenzoazepines-based compounds, particularly compounds **12** (promazine), **13** (chlorpromazine), and **14** (clomipramine) (Figure 9), as the most promising drugs. Molecular docking studies were also carried out for these compounds and demonstrated that compound **5** with a halogen substitution elicited higher binding affinity with the *T. cruzi* polyamine transporter [192].

**Figure 10:**
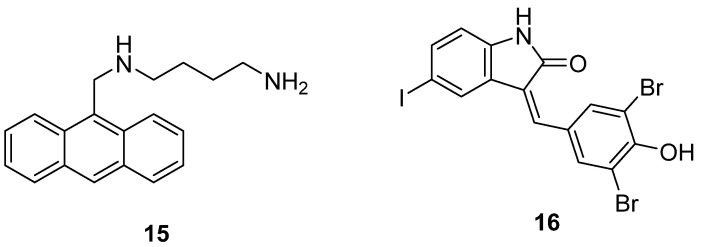
Polyamine transport system inhibitors. Ant4 (**15**), GW5074 (**16**).

Recently, other potent analogs targeting this pathway have also been identified (Figure 10), emphasizing its importance in trypanosomiasis. One such work discerned the inhibitory effect of an anthracene–putrescine analog (**15**) on *T. cruzi* with an IC_50_ of 16.97 µM against epimastigotes and 0.46 µM against trypomastigotes [193]. This research alluded to the potential of this derivative in inhibiting TcPAT12, which is believed to be the sole polyamine transporter in T. cruzi. Yet another work involved a comprehensive search to identify type-2 polyamine transporters that can potentially block polyamine transport even in higher concentrations of polyamines. In this pursuit, an oxindole-based compound (**16**) with IC_50_ of 30 µM has been identified to be capable of directly inhibiting the uptake of native polyamines such as putrescine and spermidine in human pancreatic cell lines, an attribute not seen with the use of isotretinoin [194]. This research, spurred by the absence of human polyamine transporter identification, holds promise in overcoming challenges related to the direct comparison of purported inhibitors in human cells.

The extensive research work performed on polyamine transport systems proves its necessity for the growth and survival of the protozoal parasites and in disease manifestation [64,194,195,196]. However, limited information exists regarding polyamine uptake, and only a handful of proteins have been proposed as potential polyamine transporters. In a recent discovery, a previously unknown gene ATP13A3 was pinpointed as a crucial element in the mammalian polyamine transport system (PTS) [197,198]. This gene plays a significant role in the polyamine transport deficiency observed in mutant Chinese hamster ovary cell lines generated by random mutagenesis (CHO-MG cells), a commonly utilized model for studying the mammalian PTS and potential therapeutic inhibitors of polyamine transporter. Besides this, another gene that is commonly implicated in the polyamine transport inhibition in mammalian cells is ATP13A2. This revelation raises questions on the existence as well as the involvement of related genes, such as ATP13A4 and ATP13A5 (closely related isoforms of ATP13A3), CATP-5 (transporter protein gene in *C. elegans*), and CG32000 (Drosophila gene) in the pathogens.

## 7. Conclusions

After the initial success in the early 1980s with DFMO as an irreversible ODC inhibitor against African trypanosomiasis, enthusiasm for finding new and better inhibitors of polyamine synthesis as a drug target for trypanosomatid diseases has diminished considerably. DFMO is a drug with few side effects, and in combination (NECT), it can cure late-stage infections caused by *T. b. gambiense*. However, it is not curative against *T. b. rodhesiense* or any other trypanosomatid infections. Moreover, it is a drug with serious pharmacokinetic problems that have not yet been addressed either by modifications of its structure or by formulations that improve its bioavailability. Despite its drawbacks, DFMO is on the WHO Model List of Essential Medicines. The expectations raised by DFMO inhibitors were not fulfilled in either AdoMetDC or SpdS. For the former, several interesting inhibitors have been described, although none of them went beyond the early stages of clinical trials, whereas the latter has hardly been explored. Finally, the essentiality of the trypanothione pathway and its obvious potential for selectivity against the human host have not yielded the results that were initially expected. Although hundreds of papers on TryR inhibitors have been published, the finding that many TryR inhibitors have shown off-target activity seems to indicate that TryR is not as interesting a drug target as initially expected. Without a doubt, polyamine metabolism, with all its variants, remains an interesting site of pharmacological intervention for new compounds or combinations aimed to treat these diseases, still devastating entire populations in underserved regions of the world.

## Data Availability

Not applicable.
